# A Rare Entanglement: Self-Knotting of a Nasogastric Tube

**DOI:** 10.7759/cureus.55833

**Published:** 2024-03-09

**Authors:** Shiva Shiva, Johny Lalhruaitluanga, Jyoti Singh, Amit Karnik, Awanish Kumar

**Affiliations:** 1 Surgery, King George's Medical University, Lucknow, IND

**Keywords:** ryle's tube, knot nasogastric, nasogastric tube (ngt), lariat loop, enteral feeding

## Abstract

While nasogastric intubation is a commonplace procedure characterized by its utility in enteral feeding and gastrointestinal decompression, instances of unexpected complications are relatively infrequent. Herein, we describe an unusual and rare complication, knot formation, that surfaced during routine patient care. This unique case prompts a re-evaluation of the potential complications associated with nasogastric tube insertion and offers insights into the challenges faced in its management. Through this report, we aim to contribute to the understanding of rare complications in enteral feeding practices.

## Introduction

Nasogastric tube (NGT) placement is a routine and relatively safe procedure commonly employed for gastric decompression and enteral feeding. Although relatively less invasive, it is not exempt from complications, which range from minor mucosal trauma to mortality. Complications include nasal (epistaxis, ulceration, impaction in posterior nasopharynx), laryngeal injury, esophageal (erosion and perforation), pulmonary (atelectasis, hemorrhage, pneumothorax, respiratory distress, lung abscess, isocalothorax, pleural knotted tube), tube-related (misplacement into the airway, breakdown), intravascular penetration, and even intracranial entry [1]. Tracheo-pulmonary complications occur in 0.3 to 8% of patients, with a mortality rate of 0.3% [2]. Fatal hydrothorax, pneumothorax, and empyema have also been reported as a result of NGT mispositioning [3]. ‘Esophageal bezoar’ and ‘nutrothorax’ resulting from enteral feeding via malpositioned NGT were reported by Tawfic et al. and Felipe et al. [4,5]. Kolbitsch reported a pneumothorax from a feeding tube in a patient with bilateral lung transplantation, compromising the bronchial anastomosis [6]. Granier reported the incorporation of the tube, which was inserted before thoracotomy, in a bronchial suture line following a right lower lobectomy, requiring a second thoracotomy for its removal [7]. Prolonged presence of NGT can cause necrosis of the nasal ala, ulceration, and infection of the posterior cricoid region, with subsequent dysfunction of vocal cord abduction termed nasogastric tube syndrome [8]. Very rarely, coiling the tube on itself results in ‘lariat looping’ and knot formation, which further tightens on traction during retrieval.

## Case presentation

**Figure 1:**
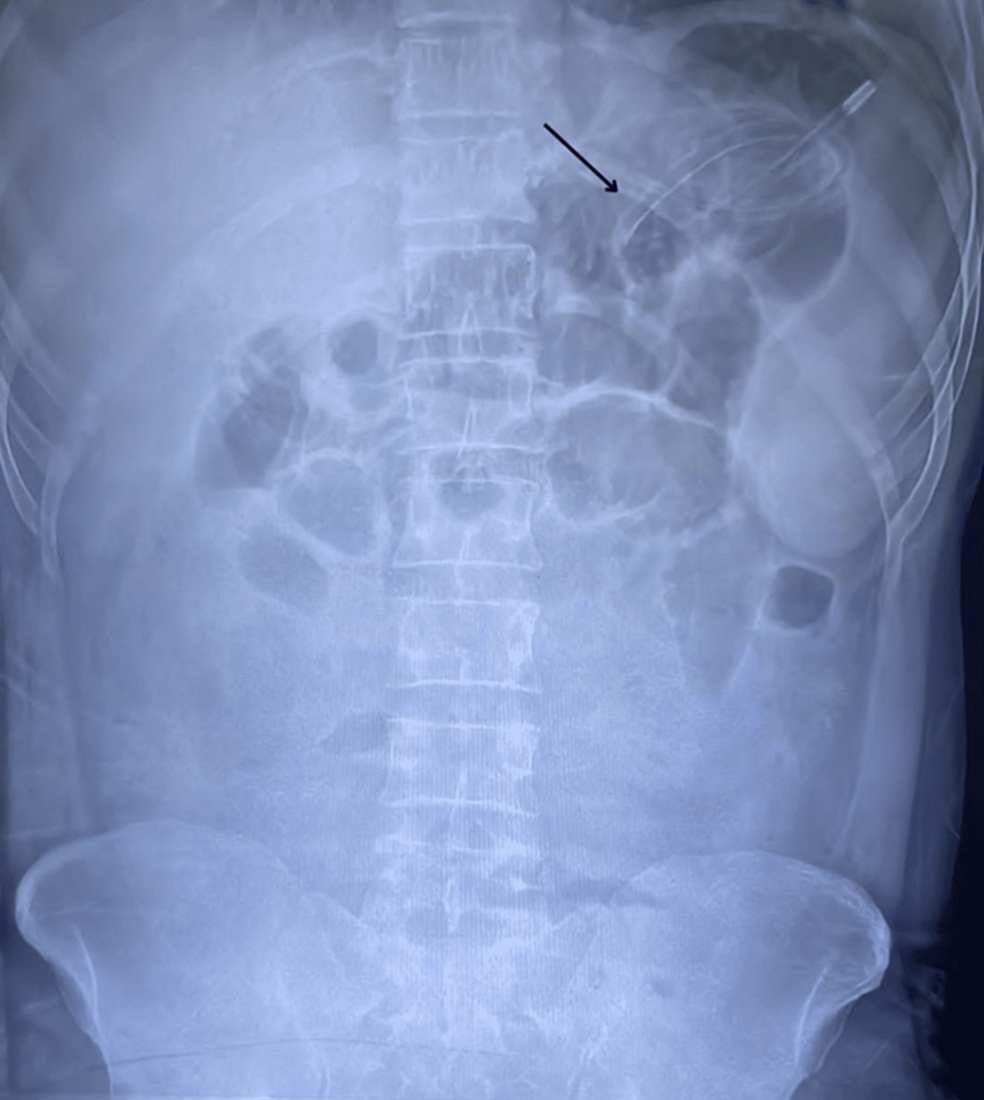
Abdominal radiograph demonstrating the loop‐forming nasogastric feeding tube (Arrow)

A 33-year-old male patient was referred to our hospital as a case of acute appendicitis with paralytic ileus. Following all aseptic precautions, a well-lubricated, single-lumen 16-French nasogastric tube was inserted blindly in the sitting position, with the neck slightly flexed to facilitate gastric drainage. The tube position was confirmed by injecting 50 cc of air through it, auscultating the epigastric area, and also by aspiration of gastric content. The tube was fixed at a 55-cm mark, with the distal end of the tube connected to a free drainage bag. The patient was managed with intravenous antibiotics, analgesics, and fluids. On day four of presentation, the patient was symptomatically relieved, was tolerating oral feeds, and the removal of the nasogastric tube was planned. While attempting removal, significant resistance was experienced near the end of the tube, and it could not be removed. The chest radiograph, when visited again, revealed a loop at the distal end of the tube (Figure 1), which probably got tightened because of the traction during the retrieval.

**Figure 2:**
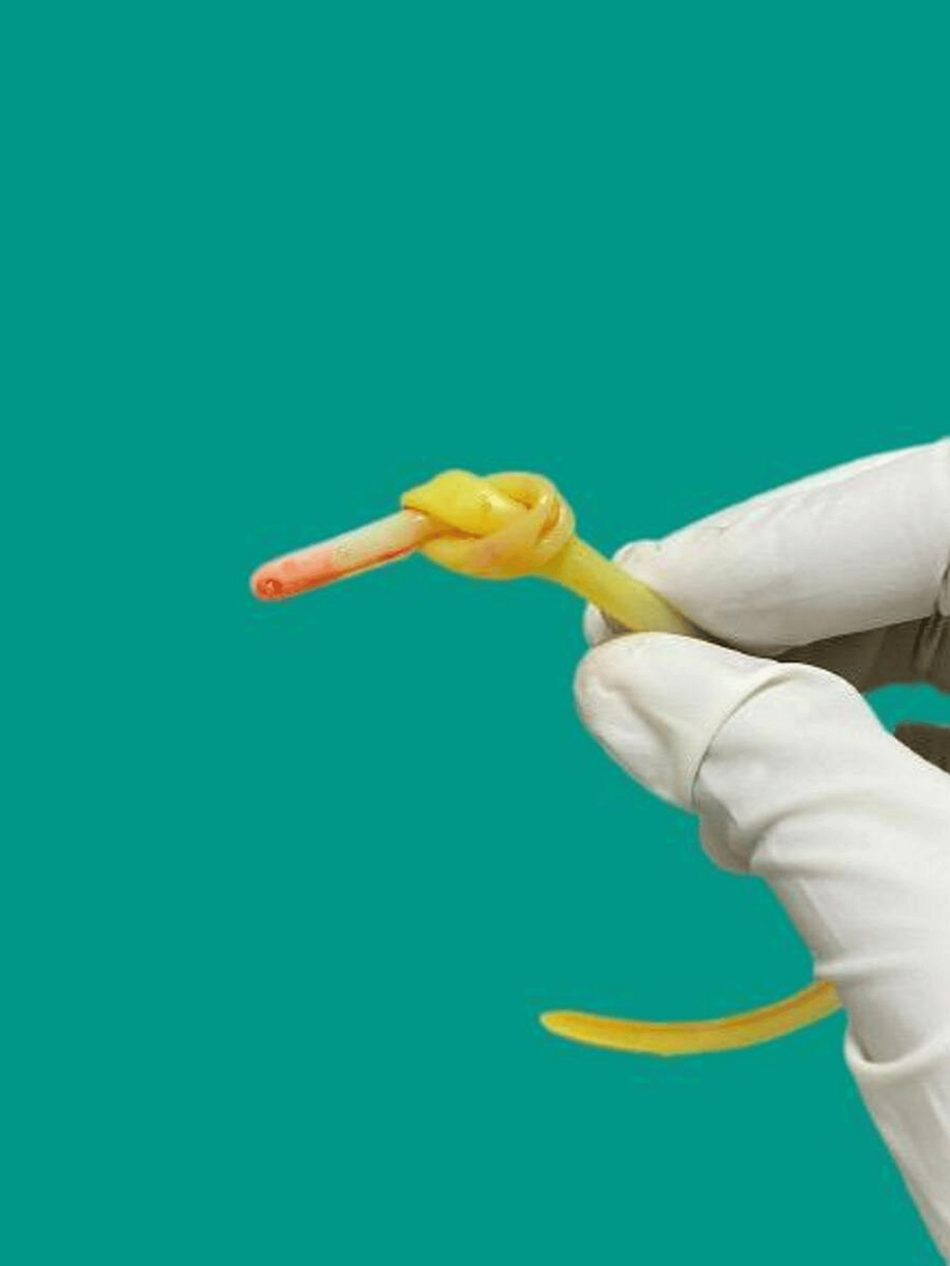
Knotted nasogastric tube after removal

Under the aid of direct laryngoscopy, the tube was pulled out gently until it was visible in the oropharynx, and the knotted end of the tube was retrieved through the mouth using Magill’s forceps and cut while the remaining length of the tube was easily delivered out through the nose. On inspection, the tube was found to have a complete knot, two cm from the distal end (Figure 2). The whole event was non-traumatic, with minimal patient discomfort.

## Discussion

Around 1.2 million NGTs are placed annually in the United States as enteral feeding is safer, more convenient, and more cost-effective [9]. Confirming tube position is vital before feeding, and the usual methods of auscultation over the epigastric area and checking the pH of the aspirate are not foolproof. Radiographic confirmation is considered to be the gold standard, but even after that, soft and smaller bore NGTs can become mispositioned after coughing, retching, or vomiting; hence, the use of more than one confirmatory method and regular rechecking are suggested [4].

Self-knotting of NGT is an extremely rare complication. Factors predisposing to knotting include narrow bore tubes, multiple manipulations during insertion, deep insertion into the stomach, a small stomach (after surgery, pediatric patients), altered mental status, decreased gag reflex, and intubated patients [1]. Knotting has also been reported, even if it is passed via a supraglottic device that has an integrated gastric channel to facilitate passage of the tube [10]. The usual mechanism of knotting is the coiling of the tube on itself, which further tightens traction during retrieval.

The occurrence of knotting can be mitigated by employing established procedures and maneuvers designed to facilitate smooth insertion. These include ensuring adequate lubrication, selecting an appropriate tube size, ensuring proper insertion length, utilizing a wide-bore tube, cooling the tube, enhancing rigidity by inserting a Fogarty catheter through a suction port, displacing the larynx forward, applying lateral neck pressure, providing direct guidance with two fingers in the mouth, and employing Magill forceps under direct laryngoscopic visualization [11]. Determining the proper tube length involves measuring the distance from the nose to the pinna and from the pinna to the xiphoid process, then adding 5cm for accurate placement.

When facing difficulty during removal, the possibility of knotting the distal end of the nasogastric tube should be considered; the tube must not be removed forcibly, and radiographs of the chest and lateral neck should be done. The best possible way to remove them is with a Magill forceps via the oral route; removal via a fiberoptic bronchoscope and esophagogastroduodenoscopy has also been reported [12,13].

## Conclusions

In conclusion, this case serves as a reminder to healthcare practitioners of the unpredictable nature of procedural complications, even in routine interventions, and sharing such instances contributes to the collective understanding of potential risks associated with nasogastric tube placement, fostering a culture of continuous improvement in procedural protocols and patient safety.
